# Reversal of *β*-Amyloid-Induced Microglial Toxicity *In Vitro* by Activation of Fpr2/3

**DOI:** 10.1155/2020/2139192

**Published:** 2020-06-13

**Authors:** Edward S. Wickstead, Husnain A. Karim, Roberta E. Manuel, Christopher S. Biggs, Stephen J. Getting, Simon McArthur

**Affiliations:** ^1^Institute of Dentistry, Barts and the London School of Medicine & Dentistry, Queen Mary, University of London, Blizard Institute, 4, Newark Street, London E1 2AT, UK; ^2^College of Liberal Arts & Sciences, University of Westminster, 115, New Cavendish Street, London W1W 6UW, UK

## Abstract

Microglial inflammatory activity is thought to be a major contributor to the pathology of neurodegenerative conditions such as Alzheimer's disease (AD), and strategies to restrain their behaviour are under active investigation. Classically, anti-inflammatory approaches are aimed at suppressing proinflammatory mediator production, but exploitation of inflammatory resolution, the endogenous process whereby an inflammatory reaction is terminated, has not been fully investigated as a therapeutic approach in AD. In this study, we sought to provide proof-of-principle that the major proresolving actor, formyl peptide receptor 2, Fpr2, could be targeted to reverse microglial activation induced by the AD-associated proinflammatory stimulus, oligomeric *β*-amyloid (oA*β*). The immortalised murine microglial cell line BV2 was employed as a model system to investigate the proresolving effects of the Fpr2 ligand QC1 upon oA*β*-induced inflammatory, oxidative, and metabolic behaviour. Cytotoxic behaviour of BV2 cells was assessed through the use of cocultures with retinoic acid-differentiated human SH-SY5Y cells. Stimulation of BV2 cells with oA*β* at 100 nM did not induce classical inflammatory marker production but did stimulate production of reactive oxygen species (ROS), an effect that could be reversed by subsequent treatment with the Fpr2 ligand QC1. Further investigation revealed that oA*β*-induced ROS production was associated with NADPH oxidase activation and a shift in BV2 cell metabolic phenotype, activating the pentose phosphate pathway and NADPH production, changes that were again reversed by QC1 treatment. Microglial oA*β*-stimulated ROS production was sufficient to induce apoptosis of bystander SH-SY5Y cells, an effect that could be prevented by QC1 treatment. In this study, we provide proof-of-concept data that indicate exploitation of the proresolving receptor Fpr2 can reverse damaging oA*β*-induced microglial activation. Future strategies that are aimed at restraining neuroinflammation in conditions such as AD should examine proresolving actors as a mechanism to harness the brain's endogenous healing pathways and limit neuroinflammatory damage.

## 1. Background

AD is the single greatest cause of dementia, affecting approximately 4% of individuals aged over 65 years and with a global disease burden of around 37 million individuals [1]. This figure is set to increase as the population ages and is expected to reach around 78 million people by 2050 [2]. There are currently no effective treatments for the condition.

Whilst the two core pathological lesions of AD, extracellular *β*-amyloid (A*β*) plaques and intraneuronal tau tangles, have long been studied, the contribution to pathology provided by neuroinflammation, and the role of the microglia in AD pathogenesis, has only recently been appreciated [3, 4]. Several lines of evidence indicate a pathological role for microglial activity: studies of genetic risk factors for idiopathic AD have identified numerous immune-related risk loci, clinical imaging studies have indicated a positive correlation between microglial activity and both A*β* load and neurodegeneration [5], and chronic neuroinflammation is considered a feature of multiple independent animal models of the disease [6]. More directly, A*β* can act as a damage-associated molecular pattern [7], stimulating microglial activation through a range of different receptors, including the receptor for advanced glycation end products, toll-like receptors, and CD36 [8].

Under normal conditions, inflammation is self-resolving, with numerous factors acting to “switch off” inflammatory processes [9]. A central actor in this process is the G protein-coupled receptor formyl peptide receptor 2 (FPR2) or its murine functional homologues Fpr2/3 [10]. Strong evidence exists for the proresolving potential of this receptor in peripheral inflammation, where it promotes neutrophil apoptosis [11] and regulates monocyte/macrophage recruitment [12, 13], phenotype [14], and behaviour [15]. Importantly, protective effects have been identified for this receptor in diverse inflammatory settings, including sepsis [16], heart failure [17], and atherosclerosis [18].

Expression of FPR2 within the brain has been reported in the endothelium and in selected hippocampal and cerebellar neurons [19], but it is also expressed by microglia [20] and is rapidly upregulated following inflammatory insult [21]. Significantly, FPR2 expression has been reported in inflammatory cells infiltrating A*β* plaques in AD [22], is involved in chemotaxis to high concentrations of A*β* [23], and has been indirectly implicated in microglial A*β* phagocytosis [24]. Given the importance of this receptor in the resolution of peripheral inflammation, we hypothesised that FPR2 agonists would be able to reverse the proinflammatory effects of A*β* upon microglia, restoring normal homeostasis.

## 2. Methods

### 2.1. Drugs and Reagents

The FPR2 agonist Quin-C1 (QC1; 4-butoxy-N-[1,4-dihydro-2-(4-methoxyphenyl)-4-oxo-3(2H)-quinazolinyl]benzamide) and antagonist WRW_4_ (Trp-Arg-Trp-Trp-Trp-Trp-NH_2_) were purchased from Tocris Ltd., UK. Isolated and purified lipopolysaccharides developed in *Escherichia coli*, serotype O111:B4, were purchased from Merck Millipore, Ltd., UK. HFIP-treated human A*β*_1-42_ peptide was purchased from JPT Peptide Technologies, Berlin, Germany.

### 2.2. A*β* Oligomerisation

HFIP-treated A*β*_1-42_ stored at -80°C in DMSO was oligomerised by dilution and vortexing in PBS followed by incubation overnight at 4°C [25]. Oligomer formation was confirmed by native tricine-SDS-polyacrylamide gel electrophoresis. Briefly, 2 *μ*g oligomeric A*β* (oA*β*) was resuspended in nondenaturing sample buffer (62.5 mM Tris-base, 25% glycerol, and 1% (*w*/*v*) Coomassie Blue R-250) and loaded onto a 10% acrylamide : bis-acrylamide gel and separated by electrophoresis alongside molecular weight markers. Gels were incubated with Coomassie stain (60 mg/l Coomassie Blue R-250 and 10% *v*/*v* acetic acid, both from Sigma-Aldrich, UK). Following 24 h destaining in 10% *v*/*v* acetic acid and 50% *v*/*v* methanol (Sigma-Aldrich, UK), gels were imaged using a ChemiDoc MP Imaging System (Bio-Rad Ltd., UK). Oligomeric A*β* migrated at approximately 35 kDa, indicating the presence of hexamers/heptamers (Supplementary Figure 1).

### 2.3. Cell Culture

The murine microglial line BV2 was a generous gift from Prof. E. Blasi (Università degli Studi di Modena e Reggio Emilia, Italy); the human neuroblastoma SH-SY5Y line was purchased from the European Collection of Authenticated Cell Cultures (ECACC, Salisbury, UK). Both lines were cultured in DMEM medium supplemented with 5% fetal calf serum and 100 *μ*M nonessential amino acids, 2 mM L-alanyl-L-glutamine, and 50 mg/ml penicillin-streptomycin (all from Thermo Fisher Scientific, UK) at 37°C in a 5% CO_2_ atmosphere. SH-SY5Y cells were differentiated to a neuron-like phenotype prior to experimentation by incubation with 10 *μ*M *trans*-retinoic acid (Sigma-Aldrich, UK) for 5 days [26].

Primary murine microglia were prepared from C57Bl/6 male mice aged 8 weeks according to our previously published protocols [27]. Cells were cultured in DMEM medium supplemented with 20% fetal calf serum and 100 *μ*M nonessential amino acids, 2 mM L-alanyl-L-glutamine, and 50 mg/ml penicillin-streptomycin (all Thermo Fisher Scientific, UK) at 37°C in a 5% CO_2_ atmosphere.

### 2.4. Reactive Oxygen Species (ROS) Assays

Total intracellular ROS production was quantified using 6-chloromethyl-2′,7′-dichlorodihydrofluorescein diacetate, acetyl ester (CM-H_2_DCFDA; Thermo Fisher Scientific, UK) according to the manufacturer's recommendations. Briefly, cells were plated at 200,000 cells/cm^2^ in phenol red-free DMEM, serum starved overnight, and preloaded with 5 *μ*M CM-H_2_DCFDA for 20 minutes at 37°C. Following removal of unbound dye, fresh phenol red-free DMEM was added and experimental treatments were begun. Following administration of treatments, cellular fluorescence was determined every 5 minutes for 1 hr at 37°C using a CLARIOstar fluorescence microplate reader (BMG Labtech, Germany) with excitation and emission filters set at 492 nm and 517 nm, respectively. Primary microglia were treated in the same way, except that due to lower numbers of cells, fluorescence was analysed at the end of 1-hour incubation periods by flow cytometry using a FACSCanto II flow cytometer (BD Biosciences, UK) equipped with a 488 nm laser and FlowJo 8.8.1 software (TreeStar Inc., FL, USA). A total of 10,000 singlet events per sample were quantified.

Mitochondrial superoxide production was quantified using the tracer MitoSOX Red (Thermo Fisher Scientific, UK) according to the manufacturer's recommendations and a loading concentration of 2.5 *μ*M. Following administration of treatments, cellular fluorescence was determined every 5 minutes for 1 hr at 37°C using a CLARIOstar fluorescence microplate reader (BMG Labtech, Germany) with excitation and emission filters set at 510 nm and 580 nm, respectively.

Hydrogen peroxide production was quantified using the ROS-Glo H_2_O_2_ Assay (Promega, Southampton, UK) according to the manufacturer's recommendations. Following experimental treatment, luminescence of cell lysates at 37°C was determined using a CLARIOstar luminescence microplate reader (BMG Labtech, Germany), in comparison to a H_2_O_2_ standard curve (0.013 *μ*M-10 mM).

### 2.5. GSH : GSSG Ratio Analysis

The ratio of reduced (GSH) to oxidised (GSSG) glutathione was determined using a commercial assay (GSH : GSSG-Glo assay, Promega, Southampton, UK) according to the manufacturer's instructions, with cells plated at 200,000 cells/cm^2^ on black-walled 96-well plates. A CLARIOstar spectrophotometer (BMG Labtech, Germany) was used to measure relative luminescence with comparison to a total glutathione standard curve (0.25 *μ*M-16 *μ*M).

### 2.6. Cytokine ELISA

Tumour necrosis factor alpha (TNF*α*) was assayed by murine-specific sandwich ELISA using commercially available kits, according to the manufacturer's protocols (Thermo Fisher Scientific, UK). A CLARIOstar spectrophotometer (BMG Labtech, Germany) was used to measure absorbance at 450 nm.

### 2.7. *E. coli* Bioparticle Phagocytosis

Microglial phagocytic capacity was determined using BODIPY FL-conjugated *Escherichia coli* (K-12 strain) bioparticles (Thermo Fisher Scientific, UK). Following experimental treatments, cells were incubated with bioparticle conjugates at a ratio of 50 particles per cell in PBS for 30 minutes at 37°C in the dark. Cells were washed, fluorescence of nonengulfed particles was quenched by the addition of 0.2% Trypan blue (Thermo Fisher Scientific, UK) for 1 min, and cellular fluorescence was determined using a FACSCanto II flow cytometer (BD Biosciences, UK) equipped with a 488 nm laser and FlowJo 8.8.1 software (TreeStar Inc., FL, USA). A total of 10,000 singlet events per sample were quantified.

### 2.8. Flow Cytometry

BV2 or SH-SY5Y cells alone or in coculture were labelled with APC-conjugated rat monoclonal anti-mouse CD11b, PE-Cy7-conjugated rat monoclonal anti-mouse CD40, or PerCP-Cy5.5-conjugated mouse monoclonal anti-human CD200 (all from BioLegend, UK) for analysis by flow cytometry. Immunofluorescence was analysed for 10,000 singlet events per sample using a FACSCanto II flow cytometer (BD Biosciences, UK); data were analysed using FlowJo 8.8.1 software (TreeStar Inc., FL, USA).

### 2.9. Annexin A5 Apoptosis Assay

SH-SY5Y cells were differentiated as described above and treated according to experimental design, either alone or in coculture with BV2 cells. Cultures were incubated in PBS, detached using a cell scraper, and incubated with FITC-conjugated Annexin A5 (0.45 *μ*g/ml in 0.01 M PBS, 0.1% bovine serum albumin, and 1 mM CaCl_2_), and in the case of cocultures, incubated with APC-conjugated rat monoclonal anti-mouse CD11b and PerCP-Cy5.5-conjugated mouse monoclonal anti-human CD200 (all BioLegend, UK) on ice in the dark for 30 min. Samples were washed and analysed by flow cytometry. Immunofluorescence was analysed for 10,000 singlet events per sample using a FACSCanto II flow cytometer (BD Biosciences, UK); data were analysed using FlowJo 8.8.1 software (TreeStar Inc., FL, USA).

### 2.10. Western Blot Analysis

Samples boiled in 6x Laemmli buffer were subjected to standard SDS-PAGE (10%) and electrophoretically blotted onto Immobilon-P polyvinylidene difluoride membranes (Merck Millipore, Ltd., UK). Total protein was quantified using Ponceau S staining (Merck Millipore, Ltd., UK) and membranes were blotted using antibodies raised against murine haem oxygenase-1 (HO-1; rabbit polyclonal, 1 : 1000, Cell Signaling Technology, Leiden, The Netherlands) or superoxide dismutase 2 (SOD2; rabbit monoclonal, 1 : 1000, Cell Signaling Technology, Leiden, The Netherlands) in Tris-buffer saline solution containing 0.1% Tween-20 and 5% (*w*/*v*) nonfat dry milk overnight at 4°C. Membranes were washed with Tris-buffer saline solution containing 0.1% Tween-20 and incubated with secondary antibody (horseradish peroxidase–conjugated goat anti-rabbit 1 : 5000; Thermo Fisher Scientific, UK) for 90 min at room temperature. Proteins were then detected using enhanced chemiluminescence detection (2.5 mM luminol, 0.4 mM p-coumaric acid, 7.56 mM H_2_O_2_ in 1 M Tris, pH 8.5) and visualised on X-ray film (Scientific Laboratory Supplies Limited, Nottingham, UK). Films were digitized and analysed using ImageJ 1.51w software (National Institutes of Health).

### 2.11. Immunofluorescence and Confocal Microscopy

Following experimental treatment, BV2 cells cultured in chambered microslides were fixed by incubation in 2% formaldehyde in PBS for 10 min at 4°C, washed, and nonspecific antibody binding was minimised by incubation for 30 min at room temperature in PBS containing 10% FCS and 0.05% Triton X-100 (all Thermo Fisher Scientific, UK). Cells were then incubated with rabbit anti-mouse p67phox monoclonal antibody (1 : 500, clone EPR5064, Abcam Ltd., Cambridge, UK) and mouse anti-mouse gp91phox monoclonal antibody (1 : 50, clone 53, BD Biosciences, UK) overnight at 4°C in PBS with 1% FCS and 0.05% Triton X-100. Cells were washed and incubated with AF488-conjugated goat anti-mouse and AF647-conjugated goat anti-rabbit secondary antibodies (both 1 : 500, Thermo Fisher Scientific, UK) in PBS with 1% FCS and 0.05% Triton X-100 at room temperature for 1 hr. Cells were washed with PBS, nuclei were defined by incubation with 180 nM DAPI in ddH_2_O for 5 min, and cells were mounted under Mowiol mounting solution. Cells were imaged using an LSM 710 Confocal Microscope (Zeiss, UK) fitted with 405 nm, 488 nm, and 647 nm lasers and a 63x oil immersion objective lens (NA 1.4 mm, working distance 0.17 mm). Images were captured with ZEN Black software (Zeiss, Cambridge, UK) and analysed with ImageJ 1.51w (National Institutes of Health, USA).

### 2.12. Glucose 6-Phosphate Dehydrogenase Activity Assay

Glucose 6-phosphate dehydrogenase (G6PD) activity was assessed using a commercial assay (Cell Signaling Technology, UK) according to the manufacturer's instructions. Following treatment according to experimental design, cells were lysed by ultrasonication (2 × 20 s at 20 kHz) in assay lysis buffer (22 mM Tris-HCl, 150 mM NaCl, 1 mM Na_2_EDTA, 1 mM EGTA, 1% Triton X-100, 20 mM sodium pyrophosphate, 25 mM sodium fluoride, 1 mM *β*-glycerophosphate, 1 mM sodium orthovanadate, 1 *μ*g/ml leupeptin, and 1 mM phenylmethylsulfonyl fluoride; pH 7.5, 4°C) using a Soniprep 150 (BMG Labtech, UK) and centrifuged at 14,000 g and 4°C for 10 min, and lysates were collected. Samples were diluted to 0.2 mg/ml in assay buffer, incubated at 37°C for 15 min with assay substrate, and fluorescence was analysed using a CLARIOstar spectrophotometer (BMG Labtech, Germany) with excitation and emission filters set at 540 nm and 590 nm, respectively.

### 2.13. Mitochondrial Function Assay

Mitochondrial function was assessed using a Seahorse XF24 Cell Mito Stress Test (Agilent Technologies Inc., California, USA) according to the manufacturer's instructions. BV2 cells plated at 2 × 10^6^ cells/cm^2^ were serum starved overnight and treated according to experimental design. Medium was replaced with Seahorse XF DMEM supplemented with 1 g/l glucose and 1 mM sodium pyruvate, pH 7.4 (Sigma-Aldrich, UK), and cells were incubated at 37°C without CO_2_ for 45 min prior to analysis of oxygen consumption rate (OCR) and extracellular acidification rate (ECAR). Basal respiration was initially determined prior to subsequent serial cellular treatments with 4 *μ*M oligomycin, 0.6 *μ*M FCCP, and 1 *μ*M rotenone/antimycin A to measure ATP production, maximal respiratory capacity, and nonmitochondrial respiration, respectively. For each treatment, readings were taken in triplicate every 5 min. Cells were lysed in RIPA buffer and protein content was assessed by Bradford's method for sample normalisation. Rates of glycolytic and oxidative ATP production were then calculated as described in [28].

### 2.14. Statistical Analysis

Sample sizes were calculated to detect differences of 15% or more with a power of 0.85, and *α* was set at 5%, calculations being informed by previously published data [27, 29]. All experimental data are presented as mean ± SEM, repeated using a minimum of *n* = 3 independent culture flasks; assays were performed in triplicate. In all cases, normality of distribution was established using the Shapiro-Wilk test, followed by analysis with two-tailed Student's *t* tests to compare two groups or, for multiple comparison analysis, one- or two-way ANOVA followed by Tukey's HSD post hoc test; a *p* < 0.05 was considered statistically significant. All statistical analysis was performed using GraphPad Prism 8 software (GraphPad Software, CA, USA).

## 3. Results

### 3.1. AD-Relevant Concentrations of A*β* Do Not Induce an Inflammatory Response in BV2 Microglia

**Figure 1:**
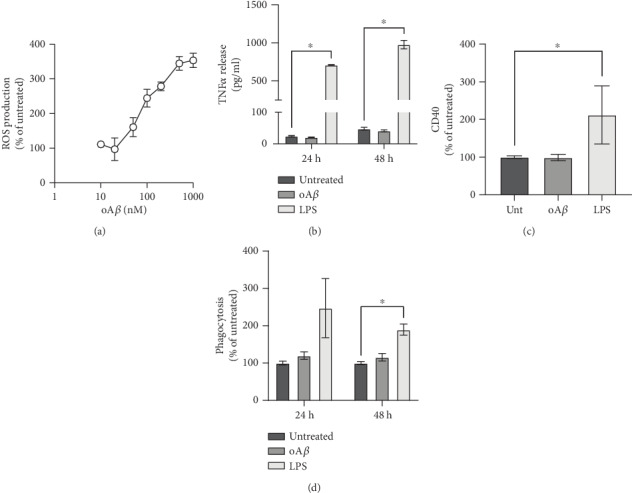
oA*β* stimulates microglial ROS production without inducing an inflammatory response. (a) Treatment with oA*β* dose-dependently induces the microglial ROS production rate over a 2 hr period. (b) Treatment of BV2 cells with 50 ng/ml LPS but not 100 nM oA*β* increased microglial TNF*α* production after 24 and 48 hr exposure. (c) Treatment for 24 hrs with 50 ng/ml LPS but not 100 nM oA*β* increased BV2 cell surface CD40 expression. (d) Neither treatment for 24 nor 48 hrs with 100 nM oA*β* affected phagocytosis by BV2 cells of heat-killed *E. coli* bacterial particles. 50 ng/ml LPS increased phagocytosis at 48 hrs only. In all cases, data are mean ± SEM of 3-6 independent cultures, assayed in triplicate. ^∗^*p* < 0.05 versus untreated cells.

Whilst many studies have investigated the toxic properties of A*β*, these have in general used micromolar concentrations of the peptide, levels which are unlikely to be achieved until the end stages of AD [30]. We sought to determine the potential of Fpr2/3 as a target to control A*β*-driven inflammation earlier in the disease process when oligomeric A*β* is found in the nanomolar range [30], hence we characterised the inflammatory response of BV2 cells to AD-relevant concentrations of A*β*. Initial studies identified a clear dose-dependent increase in BV2 cell reactive oxygen species (ROS) production upon A*β* stimulation (Figure 1(a)), with 100 nM A*β* stimulating an approximately 2.5-fold increase; this concentration of A*β* was thus used for further investigation.

In contrast to ROS production, however, 100 nM A*β* did not elicit other inflammatory changes in BV2 cells, whether assessed through production of the major inflammatory cytokine TNF*α* (Figure 1(b)), induction of the inflammatory surface phenotypic marker CD40 (Figure 1(c)), or phagocytosis of labelled *E. coli* bioparticles (Figure 1(d)). This was in marked contrast to the effects of bacterial lipopolysaccharide (LPS) which was able to evoke a clear inflammatory response from BV2 cells.

### 3.2. oA*β* Induces ROS Production through NADPH Oxidase Activation, a Response Reversed by Fpr2/3 Agonist Treatment

**Figure 2:**
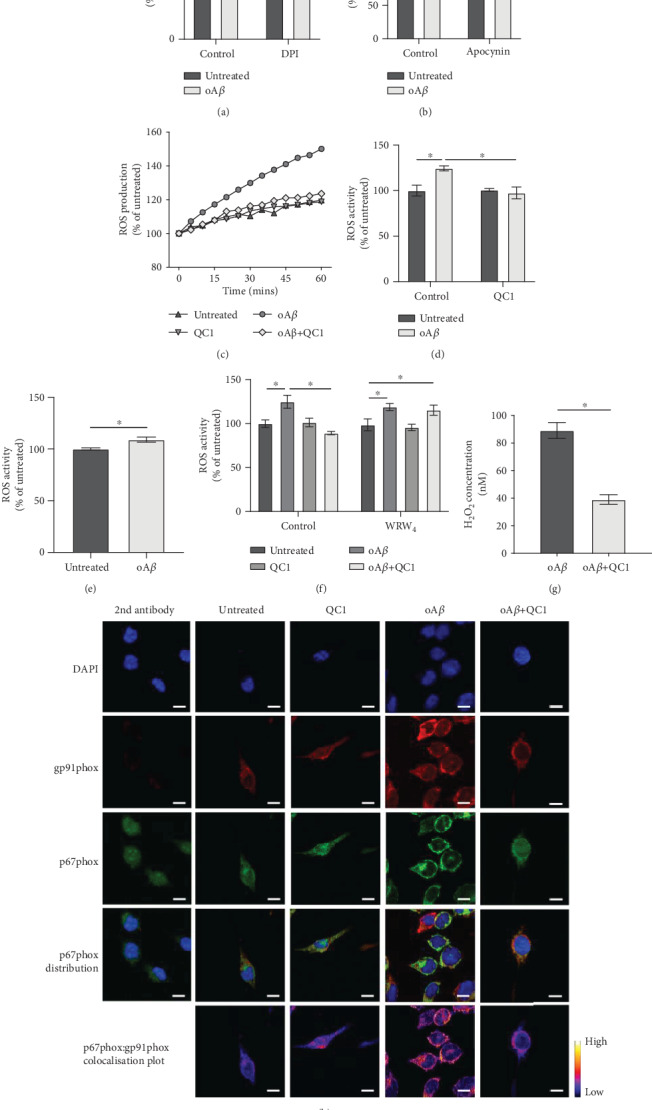
oA*β*-induced ROS production follows activation of NADPH oxidase and is reversed by subsequent Fpr2/3 stimulation. (a, b) oA*β*-induced ROS production was prevented by 10 min pretreatment with the NADPH oxidase inhibitors DPI (1 *μ*M, a) and apocynin (1 *μ*g/ml, b). (c) Representative time course of ROS production in untreated BV2 cells and cells exposed to 100 nM oA*β* with or without subsequent stimulation with 100 nM QC1 (10 min post-oA*β*). (d) Average ROS production rates for BV2 cells treated with oA*β* (100 nM, 1 hr) with or without subsequent stimulation with 100 nM QC1 (10 min post-oA*β*). (e) Treatment for 1 h of primary murine microglia from wild-type mice with 100 nM oA*β* induced significant ROS production. (f) Inclusion of the selective Fpr2/3 antagonist WRW_4_ (10 *μ*M, 10 min prior to oA*β* treatment) did not affect 100 nM oA*β*-induced ROS production but prevented the effects of subsequent treatment with QC1 (100 nM, 10 min post-oA*β*). (g) Treatment of BV2 cells for 1 h with oA*β* (100 nM) induced significant production of the ROS hydrogen peroxide, an effect blocked by subsequent treatment with QC1 (100 nM, 10 min post-oA*β*). (h) Treatment of BV2 cells for 30 min with 100 nM oA*β* stimulated colocalisation of the NADPH oxidase subunits p67phox (green) and gp91phox (red), an effect prevented by treatment with 100 nM QC1 administered 10 min post-oA*β*. Nuclei are counterstained with DAPI (blue); p67phox and gp91phox colocalisation intensity is represented by the false-colour plots. Graphical data are mean ± SEM of 3-6 independent cultures, assayed in triplicate ^∗^*p* < 0.05. Images represent cells from 3 independent cultures; scale bar = 10 *μ*m.

Microglial ROS production via the enzyme NADPH oxidase, also termed NOX2, is a key response to inflammatory stimuli, primarily serving as an antimicrobial defence mechanism [31]. There is evidence for the activation of this enzyme in AD [32], hence we investigated whether this was also the cellular source of ROS in our model. ROS production induced by stimulation with 100 nM oA*β* was sensitive to inclusion of two different NADPH oxidase inhibitors, 1 *μ*M diphenylene iodonium and 1 *μ*g/ml apocynin (Figures 2(a) and 2(b)), strongly suggesting the involvement of this enzyme. NADPH oxidase is not the only potential cellular source of ROS, however, with mitochondrial superoxide production playing a significant part in many physiological and pathological processes [33]. However, examination of BV2 cells stimulated with 100 nM oA*β* found no change in mitochondrial superoxide production over 1 hr (Supplementary Figure 2A), whereas exposure to the mitochondrial complex I inhibitor rotenone (1 *μ*M) resulted in a clear increase in mitochondrial superoxide production compared to untreated BV2 cells (Supplementary Figures 2A and 2B).

As we have previously shown that BV2 cells express murine Fpr2/3 [29], we investigated whether activation of this receptor could reverse oA*β*-induced ROS production. Treatment of cells with the Fpr2/3-specific agonist QC1 (100 nM), delivered 10 min after oA*β*-stimulation, restored ROS production to baseline levels (Figures 2(c) and 2(d)). Similarly, ROS production was detectable in primary murine microglia exposed to oA*β* (100 nM; Figure 2(e)). Moreover, oA*β*-induced ROS production from BV2 cells was sensitive to pretreatment with the Fpr2/3-specific antagonist WRW_4_ at 10 *μ*M (Figure 2(f)). Notably, production of ROS in response to oA*β* itself was not affected by WRW_4_ inclusion, indicating that oA*β* is not in this case signaling through Fpr2/3 (Figure 2(f)). Confirming these data, measurement of total cellular H_2_O_2_ revealed that whilst this species was undetectable in unstimulated cells, oA*β* treatment caused significant production, an effect reversed by treatment with QC1 (Figure 2(g)).

NADPH oxidase is a multisubunit enzyme, with its activation requiring the translocation of a p67 subunit from the cytosol to associate with the plasma membrane-bound gp91 subunit [32]. Confocal microscopic analysis of BV2 cells stimulated with 100 nM oA*β* indicated a clear appearance of colocalised p67phox and gp91phox signal at the plasma membrane of the cells, an effect that was again prevented by subsequent treatment (10 min post-oA*β*) with 100 nM QC1 (Figure 2(h)).

### 3.3. Fpr2/3 Stimulation Does Not Modify Major Cellular Antioxidant Systems

**Figure 3:**
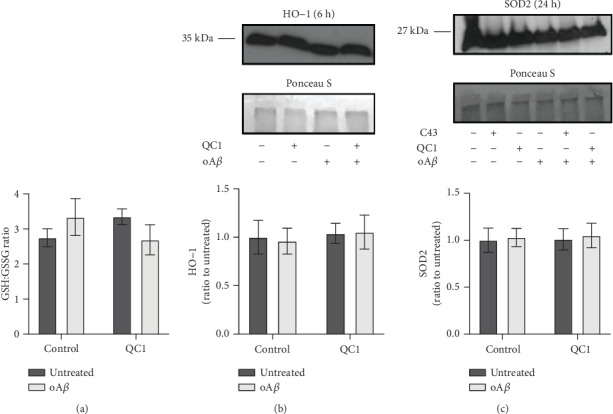
Neither treatment with oA*β* nor QC1 affected major cellular antioxidant systems. (a) The ratio of reduced (GSH) to oxidised (GSSG) glutathione within BV2 cell cytoplasm was not affected by either oA*β* (100 nM, 2 hrs) or QC1 (100 nM, 10 min post-oA*β*) administration. (b) Expression of the antioxidant enzyme haem oxygenase-1 (HO-1) was not affected by treatment with oA*β* (100 nM, 6 hrs) or QC1 (100 nM, 10 min post-oA*β*). Sample loading was normalised to Ponceau S-defined total protein content; densitometric analysis data are mean ± SEM of 3 independent cultures. (c) Expression of the antioxidant enzyme superoxide dismutase 2 (SOD2) was not affected by treatment with oA*β* (100 nM, 24 hrs) or QC1 (100 nM, 10 min post-oA*β*) or the alternative Fpr2/3 agonist Cpd43 (100 nM, 10 min post-oA*β*). All western blot analyses are representative of 3 independent cultures, with sample loading normalised to Ponceau S-defined total protein content; densitometric analysis data are mean ± SEM of 3 independent cultures, quantified in triplicate.

Whilst we have shown the Fpr2/3 agonist QC1 to reverse oA*β*-induced NADPH oxidase activation and ROS production, it is plausible that this could also be achieved through activation of intracellular antioxidant systems. However, neither the ratio of reduced to oxidised glutathione (Figure 3(a)) nor expression of the antioxidant enzymes haem oxygenase-1 (Figure 3(b)) or superoxide dismutase-2 (Figure 3(c)) was affected by treatment with either 100 nM oA*β*, 100 nM QC1, or a combination of the two. These data suggest that the ROS production-suppressing actions of Fpr2/3 activation occur through modulation at the source rather than stimulation of defensive systems.

### 3.4. Promotion of the Pentose Phosphate Pathway by oA*β* Is Reversed by Fpr2/3 Stimulation

**Figure 4:**
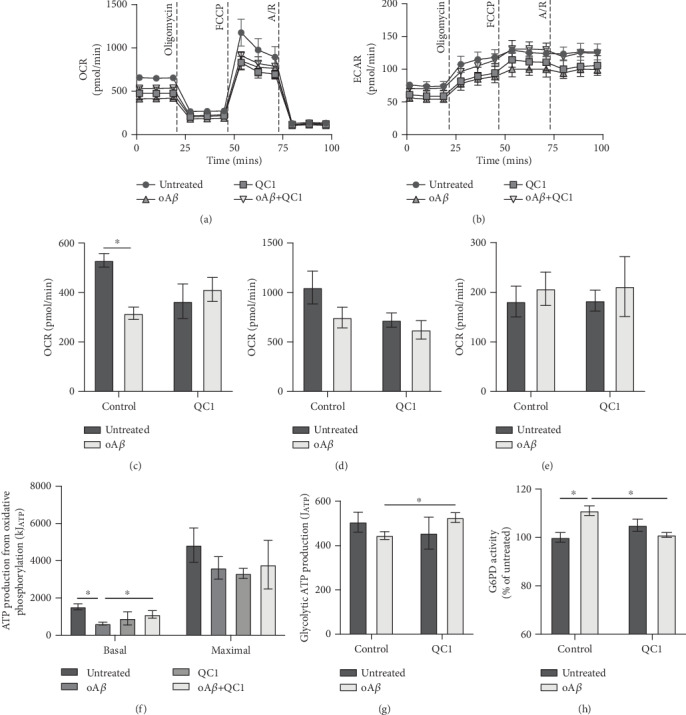
Treatment with oA*β* suppresses mitochondrial respiration and promotes activity of the pentose phosphate pathway, effects reversed by subsequent activation of Fpr2/3. (a) Typical oxygen consumption rates of untreated BV2 cells and cells treated for 24 hrs with 100 nM oA*β* with or without subsequent stimulation with QC1 (100 nM, 1 hr post-oA*β*) and administration times for oligomycin (4 *μ*M), FCCP (0.6 *μ*M), and rotenone with antimycin A (both 1 *μ*M) are indicated. (b) Typical extracellular acidification rates for untreated BV2 cells and cells treated for 24 hrs with 100 nM oA*β* with or without subsequent stimulation with QC1 (100 nM, 1 hr post-oA*β*) and administration times for oligomycin (4 *μ*M), FCCP (0.6 *μ*M), and rotenone with antimycin A (both 1 *μ*M) are indicated. (c–e) Treatment with 100 nM oA*β* for 24 hrs significantly suppressed basal metabolic rate (c), an effect that no longer reached statistical significance after QC1 treatment (100 nM, 1 hr post-oA*β*). In contrast, neither oA*β* nor QC1 treatment affected maximal respiration (d) or spare respiratory capacity (e). (f) Treatment with oA*β* (100 nM, 24 hrs) significantly suppressed basal, but not maximal, ATP production due to mitochondrial oxidative phosphorylation, an effect reversed by subsequent treatment with QC1 (100 nM, 1 hr post-oA*β*). (g) ATP generation from glycolysis was unaffected by either oA*β* or QC1 treatment. (h) Activity of the rate-determining enzyme of the pentose phosphate pathway, glucose-6-phosphate dehydrogenase (G6PD), was significantly increased by treatment with 100 nM oA*β* (24 hrs), an effect reversed by subsequent stimulation with 100 nM QC1 (1 hr post-oA*β*). All data are mean ± SEM for 3-5 independent cultures, assayed in triplicate, ^∗^*p* < 0.05.

An important aspect of immune cell activation is a change in their preferred source of metabolic energy, with inflammatory cells tending to favour glycolysis over mitochondrial oxidative phosphorylation as their primary energy source [34]. We therefore investigated how oA*β* treatment of BV2 cells would affect their metabolism through the use of the Agilent Seahorse XF Analyser. Stimulation of BV2 cells with 100 nM oA*β* significantly suppressed basal respiration without affecting either maximal respiration or spare respiratory capacity, an effect reversed by treatment with 100 nM QC1 1 hr post-oA*β* challenge (Figures 4(a)–4(e)). This change in respiration resulted in a decrease in ATP production from both oxidative phosphorylation (Figure 4(f)) and glycolysis (Figure 4(g)) upon oA*β* stimulation, an action again reversed by Fpr2/3 activation with QC1 (Figures 4(f) and 4(g)).

Production of ROS from NADPH oxidase is ultimately dependent, as its name suggests, upon a constant source of intracellular NADPH [32]. The major source of NADPH production in the cell is the pentose phosphate pathway, which siphons glucose-6-phosphate from glycolysis into the production of 6-phosphogluconate and then ribose-5-phosphate, generating NADPH in both steps [35]. As both glycolytic and mitochondrial respiratory rates were suppressed by oA*β*, we investigated whether pentose phosphate pathway activity had concomitantly risen through the measurement of the activity of the rate limiting enzyme for this pathway, glucose-6-phosphate dehydrogenase (G6PD). Treatment of BV2 cells with 100 nM oA*β* for 24 hrs caused a significant increase in G6PD activity, an effect that was reversed to baseline upon subsequent treatment with 100 nM QC1 (Figure 4(h)), confirming the importance of this pentose phosphate pathway shunt in the response to oA*β*.

### 3.5. oA*β* Stimulated ROS Production Is Responsible for Microglial-Mediated Neuronal Toxicity and Can Be Reversed by Fpr2/3 Activation

**Figure 5:**
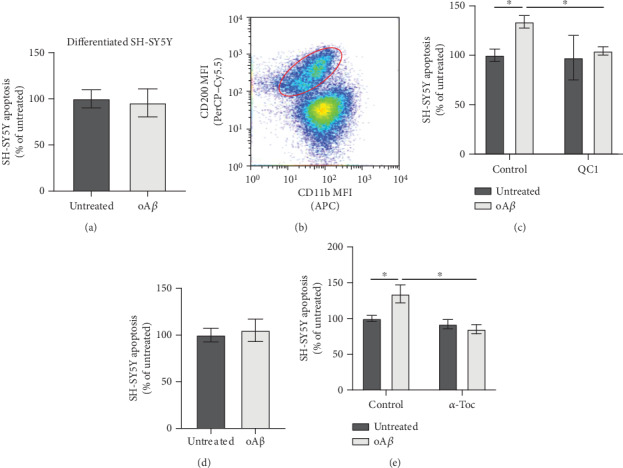
Treatment with oA*β* induces differentiated SH-SY5Y neuronal apoptosis only in the presence of microglia, acting through Fpr2/3-sensitive ROS release. (a) Treatment with oA*β* (100 nM, 48 hrs) had no effect on *trans*-retinoic acid- (tRA-) differentiated SH-SY5Y cell viability; data are mean ± SEM of 5 independent cultures, assayed in triplicate. (b) Separation of tRA-differentiated SH-SY5Y neurons from BV2 cells grown in coculture on the basis of differential CD200 and CD11b expression; plot is representative of 3 independent cultures. (c) Treatment of cocultures of BV2 and tRA-differentiated SH-SY5Y neurons with oA*β* (100 nM, 48 hrs) induces significant SH-SY5Y apoptosis, an effect prevented by subsequent treatment with QC1 (100 nM, 10 min post-oA*β*). (d) Conditioned medium from BV2 cells treated or not with 100 nM oA*β* (24 hrs) had no effect on tRA-differentiated SH-SY5Y neuronal apoptosis following exposure for 48 hrs. (e) Inclusion of the antioxidant *α*-tocopherol (10 *μ*M) in cocultures of BV2 cells and tRA-differentiated SH-SY5Y neurons prevented oA*β*-induced (100 nM, 48 hrs) neuronal apoptosis; data are mean ± SEM for 3-6 independent cultures, assayed in triplicate, ^∗^*p* < 0.05.

Production of ROS by immune cells is primarily for the purpose of killing invading pathogens. In the context of AD, however, where no infectious agent has been discovered, production of ROS may well damage bystander neurons, contributing to neurodegeneration. To investigate the relationship between oA*β*-triggered microglial ROS production and neuronal health, we employed an *in vitro* coculture model using BV2 cells and *trans*-retinoic acid-differentiated SH-SY5Y cells. Initial experiments revealed that 100 nM oA*β* showed no direct toxicity to differentiated SH-SY5Y cells even after exposure for 24 hrs (Figure 5(a)). However, administration of oA*β* to cocultures significantly and selectively enhanced apoptosis of SH-SY5Y cells (Figures 5(b) and 5(c)) without affecting BV2 cell survival (Figure 5(c)), an effect that was notably prevented by treatment with 100 nM QC1 1 hr after oA*β* exposure. Notably, differentiated SH-SY5Y cells did not express Fpr2/3 (Supplementary Figure 3). Confirming that either direct contact or short-lived secretory factors were responsible for BV2 cell-mediated toxicity, apoptosis of SH-SY5Y cells was not induced following treatment with conditioned medium from oA*β*-stimulated BV2 cultures (Figure 5(d)). Finally, to test whether BV2 cell ROS production was the mediating agent for SH-SY5Y cytotoxicity, the experiment was repeated in the presence of the antioxidant molecule *α*-tocopherol (10 *μ*M). Inclusion of this antioxidant prevented SH-SY5Y apoptosis in cocultures treated with oA*β*, indicating a direct mediatory role of microglial ROS production.

## 4. Discussion

Despite over 300 clinical trials having been performed targeting either of the proposed toxic mediators in AD, A*β* and hyperphosphorylated tau, we do not as yet have any successful therapeutic approaches for the disease. This suggests that, at the least, these two proteins cannot be the sole factors driving the disease [36]. Increasingly, the role of neuroinflammation and the behaviour of microglia in AD has come under investigation [3, 37], an approach given further impetus by reports that ablation of microglia can halt brain atrophy in murine models of A*β*-driven disease [38] and tauopathy [39]. Microglial activation can be both beneficial and damaging, hence strategies that can control excessive inflammatory activity and promote a proresolving phenotype may be of great potential for therapeutic use. In this study, we have used an *in vitro* cellular model to provide proof-of-principle evidence for the targeting of the proresolving receptor Fpr2/3 as a mechanism to restrain microglial behaviour and limit the ability of these cells to damage bystander neurons.

Here, we report that alongside its well-characterised function in resolving inflammation and efferocytosis [40], Fpr2/3 activation can reverse oA*β*-induced ROS production through deactivation of NADPH oxidase activity. Activation of microglial NADPH oxidase by oA*β* is well supported [41, 42] and may be critical in triggering neuroinflammation, given the damaging effects of oxidative stress for neurons, as has been reported in traumatic brain injury [43]. Future work will determine whether the *in vitro* findings we report here can be extended to the *in vivo* situation, but if so, they suggest that the use of Fpr2/3 agonists capable of reversing NADPH oxidase activation may be of therapeutic potential for AD.

The effects of Fpr2/3 activation upon oA*β*-induced ROS production are mirrored by changes in microglial metabolic phenotype. The importance of cellular metabolism in regulating immune cell phenotype has become increasingly evident over the past few years, with a shift from mitochondrial respiration to a glycolysis-dominant metabolism being closely associated with a proinflammatory phenotype [34]. Metabolic changes in AD are well supported [44], but the relationship between these changes and disease pathology is unclear. In the current study, exposure of microglia to oA*β* suppressed mitochondrial respiration, but rather than being accompanied by changes to glycolysis, it was associated with a significant diversion of glucose to the pentose phosphate pathway. Presumably, this was due to increased NADPH demand associated with NADPH oxidase-driven ROS production, as seen in peripheral macrophages [45]. Notably, Fpr2/3 agonist treatment was able to reverse the effects of oA*β* on microglial metabolism, targeting both the pentose phosphate pathway and the mitochondria. This data adds to the increasing evidence suggesting that Fpr2/3 not only suppresses proinflammatory mediator production [16, 46] but also aids in the regulation of the underlying metabolic changes that occur in activated immune cells, as we have recently shown in peripheral macrophages [14]. Importantly, microglia rapidly upregulate Fpr2/3 expression following inflammatory insult [47], and whilst the effects of its stimulation on neuroinflammation can be agonist dependent [48], selective Fpr2/3 activation contributes to neuroinflammatory resolution in a murine model of AD [46].

However, a notable finding of the current work is that we were unable to detect evidence for an oA*β*-induced microglial inflammatory response, in contrast to previous *in vitro* studies [24, 47, 49]. This does not appear to be due to a deficiency in the BV2 cells themselves, as stimulation with bacterial lipopolysaccharide was still able to trigger a potent inflammatory response. Notably, studies that report proinflammatory effects of oA*β* have commonly used micromolar concentrations of the peptide, several orders of magnitude greater than levels reported to occur in the human brain in AD [30], at least in early stages of the disease. Local accumulations of a variety of A*β* forms, including oligomeric and fibrillar, may exert further toxic effects, but given the temporal discrepancy between A*β* plaque and symptom appearance in humans [50], this may not be as relevant to the initiation stages of the disease. Moreover, this mismatch suggests that the direct proinflammatory effects of oA*β* seen *in vitro* may not be fully recapitulated *in vivo*, although this requires further validation. Nevertheless, oA*β* was clearly able to induce ROS production at levels capable of damaging bystander cells, which if replicated *in vivo* may be a driving factor in the ongoing neuronal damage and secondary neuroinflammation seen in AD. Production of ROS is far from the sole damaging effect of oA*β* in the brain, as is borne out by the, at best, equivocal results from clinical trials of antioxidants in AD [49, 51]. Nevertheless, targeting a receptor with the potential to suppress ROS production, restore microglial metabolic homeostasis, and promote resolution, as is the case for Fpr2/3, has significant potential for therapeutic development.

The goal of this study was to provide proof-of-principle evidence that exploitation of human FPR2 may hold promise as a therapeutic target for AD research. Evidently, there are limitations in how far the current study should be interpreted, with a particular need for further *in vivo* validation of our findings. Nonetheless, the data presented do suggest that this receptor would be a suitable target for antioxidative and metabolic therapeutic development for further AD research, particularly as the effects of Fpr2/3 stimulation were apparent when stimulation occurred after oA*β* treatment. This study therefore adds novel insights into the role of Fpr2/3 in modulating microglial oxidative stress and metabolism, holding promise for select Fpr2/3 agonists as potent and effective treatment options for inflammatory disease [9].

## 5. Conclusions

This study has identified that although pathologically relevant concentrations of oA*β* do not appear to directly stimulate an inflammatory microglial phenotype, oA*β* at these levels is a potent activator of microglial ROS production via NADPH oxidase, consequently altering metabolic phenotype. Moreover, activation of the proresolving receptor Fpr2/3 was able to reverse these oA*β*-induced changes and protect bystander neurons from damage. These data suggest that manipulation of Fpr2/3 may be an important target for future therapeutic development in neuroinflammatory conditions such as Alzheimer's disease.

## Data Availability

Data sharing is not applicable to this article as no datasets were generated or analysed during the current study.

## Abbreviations

AD: Alzheimer's disease
FPR2: Human formyl peptide receptor 2
Fpr2/3: Urine formyl peptide receptors 2/3
GSH: Reduced glutathione
GSSG: Oxidised glutathione
oA*β*: Oligomeric *β*-amyloid 1-42
ROS: Reactive oxygen species
TNF*α*: Tumour necrosis factor alpha
WRW_4_: Trp-Arg-Trp-Trp-Trp-Trp.

## Supplementary Materials

Supplementary Materials Supplementary Figure 1: approximate molecular weight of A*β*1-42 oligomers following polyacrylamide gel electrophoresis under nondenaturing conditions. As monomeric A*β*1-42 has a molecular weight of 4.51 kDa, the apparent molecular weight of approximately 35 kDa suggests that oA*β* species were hexamers/heptamers. Supplementary Figure 2: mitochondrial ROS production is not stimulated in microglia by oA*β* treatment. (A) Representative time course of mitochondrial superoxide production in untreated BV2 cells and cells exposed to 100 nM oA*β*, 100 nM QC1, or 1 *μ*M rotenone for 1 hr. (B) Average mitochondrial superoxide production rates for untreated BV2 cells and cells treated with oA*β* (100 nM, 1 hr), QC1 (100 nM, 1 hr), or rotenone (ROT; 1 *μ*M, 1 hr); data are mean ± SEM of 4 independent cultures, assayed in triplicate, ^∗^*p* < 0.05. Supplementary Figure 3: FPR2 is not expressed by SH-SY5Y cells. (A) Representative forward scatter-side scatter histogram plot and gating strategy. (B) Representative histogram plot to exclude cell doublets. (C) Histogram of relative FPR2 staining. Neither naïve or *trans*-retinoic acid induced differentiated SH-SY5Y cells expressed FPR2.
