# Supplementary figures and images for: Reversal of β-Amyloid-Induced Microglial Toxicity In Vitro by Activation of Fpr2/3
Source: Oxid Med Cell Longev. 2020 Jun 13;2020:2139192. doi: 10.1155/2020/2139192 (PMC7313167; doi:10.1155/2020/2139192)

Supplementary Figure 1

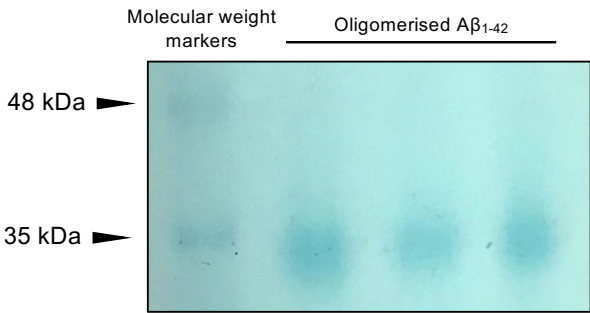

Supplementary Figure 2

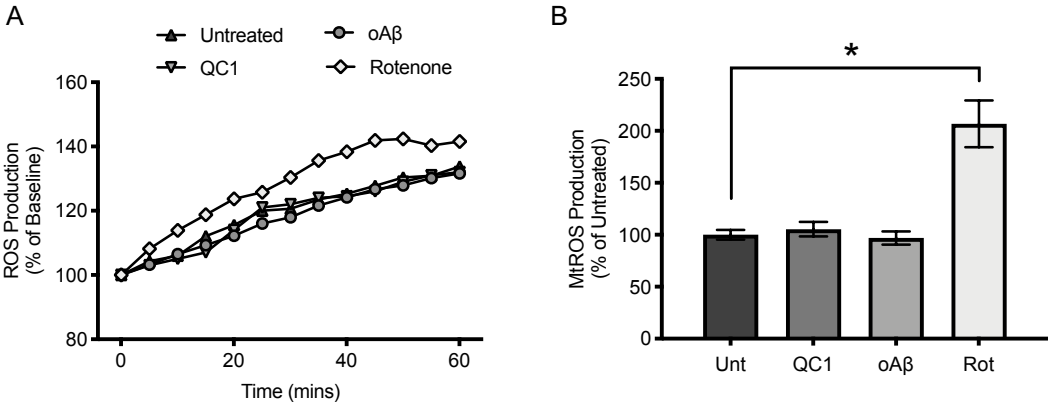

Supplementary Figure 3

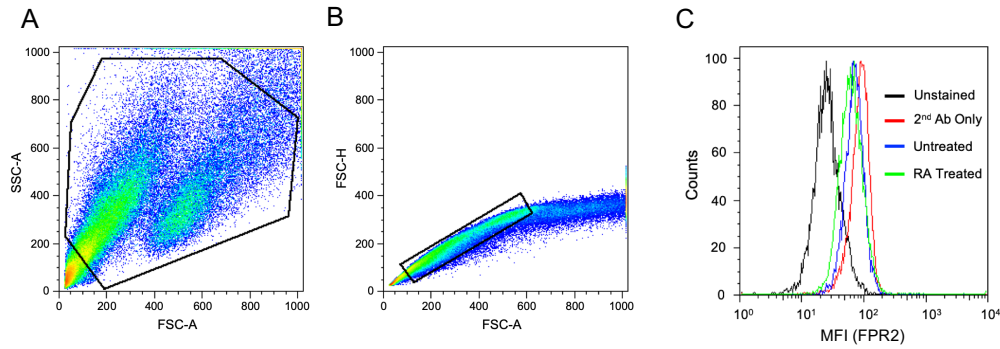

Supplement: Supplementary Materials — Supplementary Figure 1: approximate molecular weight of Aβ1-42 oligomers following polyacrylamide gel electrophoresis under nondenaturing conditions. As monomeric Aβ1-42 has a molecular weight of 4.51 kDa, the apparent molecular weight of approximately 35 kDa suggests that oAβ species were hexamers/heptamers. Supplementary Figure 2: mitochondrial ROS production is not stimulated in microglia by oAβ treatment. (A) Representative time course of mitochondrial superoxide production in untreated BV2 cells and cells exposed to 100 nM oAβ, 100 nM QC1, or 1 μM rotenone for 1 hr. (B) Average mitochondrial superoxide production rates for untreated BV2 cells and cells treated with oAβ (100 nM, 1 hr), QC1 (100 nM, 1 hr), or rotenone (ROT; 1 μM, 1 hr); data are mean ± SEM of 4 independent cultures, assayed in triplicate, ∗p < 0.05. Supplementary Figure 3: FPR2 is not expressed by SH-SY5Y cells. (A) Representative forward scatter-side scatter histogram plot and gating strategy. (B) Representative histogram plot to exclude cell doublets. (C) Histogram of relative FPR2 staining. Neither naïve or trans-retinoic acid induced differentiated SH-SY5Y cells expressed FPR2. [file 2139192.f1.pdf]
